# Targeted Next-Generation Sequencing of Korean Patients With Developmental Delay and/or Intellectual Disability

**DOI:** 10.3389/fped.2018.00391

**Published:** 2018-12-17

**Authors:** Ji Yoon Han, Ja Hyun Jang, Joonhong Park, In Goo Lee

**Affiliations:** ^1^Department of Pediatrics, College of Medicine, The Catholic University of Korea, Seoul, South Korea; ^2^Department of Laboratory Medicine, Green Cross Genome, Yongin, South Korea; ^3^Department of Laboratory Medicine, College of Medicine, The Catholic University of Korea, Seoul, South Korea

**Keywords:** developmental delay, intellectual disability, next-generation sequencing, targeted gene panel, mutation

## Abstract

**Background:** Differential diagnosis of developmental delay (DD) and/or intellectual disability (ID) is challenging because of the diversity of phenotypic manifestations as DD/ID patients usually have combined congenital malformations, autism-spectrum disorders, and/or seizure disorder. Thus, unbiased genomic approaches are needed to discover genetic alterations leading to DD and/or ID.

**Objective:** The aim of this study was to investigate the clinical usefulness of targeted next-generation sequencing (NGS) to investigate genetic causes in 35 Korean patients with unexplained DD/ID.

**Methods:** Targeted next-generation sequencing (NGS) using the TruSight One Panel was analyzed in 35 patients with unexplained DD/ID. Sanger sequencing was used to confirm candidate variants, and to define genetic inheritance mode of candidate variant as familial segregation testing.

**Results:** Of 35 patients with DD and/or ID, 10 were found to have underlying genetic etiology and carried X-linked recessive inheritance of *ZDHHC9* or autosomal dominant inheritance of *SMARCB1, CHD8, LAMA5, NSD1, PAX6, CACNA1H, MBD5, FOXP1*, or *KCNK18* mutations. No autosomal recessive inherited mutation was identified in this study. As a result, the diagnostic yield of DD/ID by targeted NGS was 29% (10/35), mostly involving may be *de novo* mutation present in the proband only. A total of seven may be *de novo* mutations, one paternally inherited, and one maternally inherited mutations that had been reported previously to concede the genetic pathogenesis as known DD and/or ID genes were found in nine patients with available inheritance pattern except *LAMA5*. Mutations in nine causative genes were detected in patients with similar DD/ID phenotypes in the OMIM database, providing support for genetic evidence as the cause of DD and/or ID.

**Conclusion:** Targeted NGS through singleton analysis with phenotype-first approaches was able to explain 10 out of 35 DD/ID cases. However, the excavation of plausible genetic causes may be *de novo*, and X-linked disease-causative variants in DD/ID-associated genes requires further genetic analysis.

## Introduction

Developmental delay (DD) is determined as a substantial delay in two or more of the following developmental domains: cognition, social/personal development, speech/language, gross/fine motor, or activities of daily living (1). Intellectual disability (ID), that is also suggested to as mental retardation or learning disability affects about 1–3% of the worldwide population of DD/ID (2). A clinical diagnosis of ID is conventionally defined as an IQ < 70 and significant difficulties in activities of general living. All children with a DD and/or ID should undergo multidimensional evaluation to establish the etiology of the disability. During early childhood, diagnosis is based on DDs; however, children with different non-syndromic forms of ID are difficult to clinically distinguish (3). Genetic causes include chromosomal aberrations and gene mutations, but genetic etiology remains unknown for about 50% of patients with DD/ID. Differential diagnosis of DD/ID is challenging because of the diversity of phenotypic manifestations as DD/ID patients usually have combined congenital malformations, autism-spectrum disorders, and/or seizure disorder (4). Genetic diagnosis can give us information on clinical outcome, prevent further superfluous invasive testing, and lead to tailored treatments (5). Family members can learn the risk of possible prenatal diagnosis, disease susceptibility, and recurrence, providing knowledge and potential psychological relief (6). Without diagnostic suspicions based on clinical presentation, unbiased genomic approaches are needed to discover genetic alterations leading to DD and/or ID (7–9).

Here, the aim of this study was to investigate the clinical usefulness of targeted next-generation sequencing (NGS) to investigate genetic causes in 35 Korean patients with unexplained DD/ID. An etiologic diagnosis for these patients had not been defined in spite of extensive clinical and genetic evaluations.

## Patients, Materials, and Methods

### Patients

Based on medical record review, we enrolled a total of 35 unrelated patients (22 males and 13 females) with unexplained DD/ID and their biological parents (trios) referred to the Department of Pediatrics, Daejeon St. Mary's Hospital (Daejeon, Republic of Korea) between January 2015 and March 2017. This study was approved by the Institutional Review Board of The Catholic University of Korea (No. DC18RESI0049). All studied patients and family members provided written informed consent for clinical and genetic testing. Consent for publication of medical information and sequencing data was obtained from the participants' parents. The parents of the children who participated in this study provided written consent on behalf of their children. Patients comprised syndromic and non-syndromic DD/ID. Before recruitment, each patient had received an extensive diagnostic evaluation such as metabolic test, fragile X syndrome gene test, hearing tests, and ophthalmologic evaluation. Serum and urine metabolic screening was evaluated whenever indicated, but these estimations had not resolved an etiologic diagnosis. Medical data were described following a standardized clinical record outlining prenatal history, neurological and behavioral disorders, and developmental history. DD was defined as >2 standard deviations below age-matched peers in two or more domains: cognition, social/personal development, speech/language, gross/fine motor, or activities of daily living in the age of ≤ 6 years. ID severity was assessed using the Wechsler Intelligence Test or Bayley Scales of Infant Development (3rd edition) by a neurological pediatrician upon clinical evaluation. ID is defined by an intelligence quotient (IQ) of ≤ 70, with deficits in ≥2 behaviors associated to adaptive functional originating in the age of < 18 years. Recurrent deletions and duplications, also defined as copy number variants (CNVs), are crucial contributors to DD/ID with complex inheritance (10). The use of chromosomal microarray (CMA) instead of G-band karyotyping has been strongly recommended as the first-tier cytogenetic testing for patients with DD/ID (11). Thus, prior to targeted NGS, CMA was conducted using a SurePrint G3 Human CGH þ SNP microarray 4 × 180 k Kit (Agilent Technologies, Inc., Santa Clara, CA) with direct sequencing for causative genes.

### Targeted Next-Generation Sequencing and Variant Annotation

Pure genomic DNA was isolated from peripheral blood using the Gentra Puregene blood kit (Qiagen Inc., Valencia, CA) according to the manufacturer's protocols. Library preparation was carried out using a TruSight One Sequencing Panel (Illumina, Inc., San Diego, CA) to enrich a 12 Mb region covering 4813 genes with clinical properness. Massively parallel sequencing was conducted on an Illumina HiSeq2000 sequencer (Illumina, Inc.). Sequence reads were mapped to human reference genome hg19 with Burrow-Wheeler Aligner 0.7.12. Read duplicates were eliminated using Picard-tools 1.96. Base-quality recalibration and local realignment were carried out using Genome Analysis Tool Kit 3.5 from the Broad Institute according to GATK's best practice guidelines for germline SNP & Indel discovery in whole genome and exome sequences. Variants were called by GATK HaplotypeCaller was used to call variants and Variant Effect Predictor and dbNSFP 2.4 (12) were used to annotate variants and estimate their functional prediction.

### Candidate Variant Prioritization

No (likely) pathogenic variants listed in the NCBI dbSNP 143 were excluded, along with variants which showed low read depth (<30×) or poor base quality score (Phred quality score <20). Variant prioritization was progressed according to the following criteria: choice of candidate genes associated to clinical manifestation, pathogenic impact of the *in-silico* prediction on gene function, of conservation change of affected amino acid, and allele frequency reported in the literature and public sequence databases such as ClinVar (https://www.ncbi.nlm.nih.gov/clinvar/), 1,000 genomes phase3 database (http://phase3browser.1000genomes.org/index.html), Exome Variant Server (EVS, http://evs.gs.washington.edu/EVS/), Exome Aggregation Consortium (http://exac.broadinstitute.org), and Human Gene Mutation Database (HGMD, http://www.hgmd.cf.ac.uk/ac/all.php). To eliminate benign variants not reported in western populations, variants of uncertain significance were also searched in 622 Korean populations based on the Korean Reference Genome Database (KRGDB, http://152.99.75.168/KRGDB/). Non-synonymous variants were considered to affect candidate genes which had not been previously implicated in DD/ID when the variant was predicted to be (likely) pathogenic by the most of prespecified *in-silico* analyses. Protein structure/function prediction and evolutionary conservation were estimated by various prediction tools.

### Sanger Sequencing Validation

Sanger sequencing was used to confirm candidate variants, and to define genetic inheritance mode of candidate variant as familial segregation testing. All candidate variants were sequenced bidirectionally with ABI PRISM 3.1 Big Dye terminator chemistry (Applied Biosystems, Foster City, CA). Sequencing products were resolved on an ABI PRISM 3130XL sequencer (Applied Biosystems), and chromatograms were analyzed with Sequencher 4.9 (Gene Codes, Ann Arbor, MI).

## Results

Of 35 patients with DD and/or ID, 10 were found to have underlying genetic etiology and carried X-linked recessive inheritance of *ZDHHC9* or autosomal dominant inheritance of *SMARCB1, CHD8, LAMA5, NSD1, PAX6, CACNA1H, MBD5, FOXP1*, or *KCNK18* mutations. No autosomal recessive inherited mutation was identified in this study. As a result, the diagnostic yield of DD/ID by targeted NGS was 29% (10/35), mostly involving may be *de novo* mutation present in the proband only. A total of seven may be *de novo* mutations, one paternally inherited, and one maternally inherited mutations that had been reported previously to concede the genetic pathogenesis as known DD and/or ID genes except *LAMA5* were found in nine patients with available inheritance pattern. Mutations in nine causative genes were detected in patients with similar DD/ID phenotypes in the OMIM database, providing support for genetic evidence as the cause of DD and/or ID. The mean age was 7.9 ± 4.8 years, and male-to-female ratio was 7 to 3. All patients had comorbidities: 3 epilepsy, 3 autism spectrum disorder (ASD), 3 hypotonia, 1 attention deficit hyperactivity disorder (ADHD), 1 obesity, 1 overgrowth, 1 aniridria, 1 ventricular septal defect (VSD), and 1 migraine with brainstem aura. Detailed clinical manifestations of these 10 patients are summarized in Table 1. Because more than half of disease-related mutations (6/10) are missense mutations, functional predictions and conservation scores for rare missense variants identified in this study were estimated to discriminate these pathogenicities using various *in-silico* analysis tools (Table 2).

**Table 1:** Clinical manifestations and candidate mutations in the patients with developmental delay and/or intellectual disability.

**Patients**	**Age**	**Clinical manifestations**				**Gene**	**Base change**	**AA change**	**# OMIM**	**Inheritance**
		**DD**	**ID**	**Epilepsy**	**Other**				
46_S11	1–5	P	nd	N	Hypotonia	*ZDHHC9*	c.286C>T	p.Arg96Trp	# 300799	*de novo*
88_S3	1–5	P	nd	N	Hypotonia	*SMARCB1*	c.31G>A	p.Gly11Arg	# 614608	*de novo*
47_S3	10–15	P	P	N	Obesity, ADHD	*CHD8*	c.4651C>T	p.Arg1551Cys	# 615032	*de novo*
35_S11	1–5	P	nd	P	Hypotonia	*LAMA5*	c.10828+1G>A		nd	*de novo*
69_S7	1–5	P	P	N	Overgrowth	*NSD1*	c.1789G>T	p.Glu866*	# 117550	*de novo*
41_S5	10–15	P	P	N	Aniridria, ASD	*PAX6*	c.19G>T	p.Gly7*	# 106210	*de novo*
36_S4	10–15	P	P	P	VSD	*CACNA1H*	c.5675G>A	p.Arg1892His	# 611942	*de novo*
46_S3	5–10	P	P	P	ASD	*MBD5*	c.254_255delGA	p.Arg85Asnfs*6	# 156200	Paternal
38_S2	5–10	P	P	N	ASD	*FOXP1*	c.155C>T	p.Ala52Val	# 613670	*de novo*
108_S9	10–15	N	P	N	Migraine	*KCNK18*	c.301T>C	p.Trp101Arg	# 613656	Maternal

*AA, amino acid; rs ID, reference SNP number; # OMIM, online mendelian inheritance in man databases; DD, developmental delay; ID, intellectual disability; P, positive; N, negative; nd, not determined; ADHD, attention deficit hyperactivity disorder; ASD, autism spectrum disorder; VSD, ventricular septal defect*.

**Table 2:** Results of *in-silico* analysis for the candidate missense mutations in the patients with developmental delay and/or intellectual disability.

**Patients**	**46_S11**	**88_S3**	**47_S3**	**36_S4**	**38_S2**	**108_S9**
Gene	*ZDHHC9*	*SMARCB1*	*CHD8*	*CACNA1H*	*FOXP1*	*KCNK18*
Accession ID	NM_016032.3	NM_003073.3	NM_020920.3	NM_021098.2	NM_032682.5	NM_181840.1
AA Change	p.Arg96Trp	p.Gly11Arg	p.Arg1551Cys	p.Arg1892His	p.Ala52Val	p.Trp101Arg
**FUNCTIONAL PREDICTIONS**						
SIFT	D (0)	D (0.001)	D (0)	T (0.12)	T (0.639)	D (0.001)
Polyphen2	D (1)	D (1)	D (1)	P (0.696)	D (0.972)	D (1)
LRT	D (0)	N (0)	D (0)	N (0)	N (0.001)	D (0)
MutationTaster	D (1)	D (1)	D (1)	N (1)	D (0.743)	D (1)
MutationAssessor	H (4.24)	M (2.505)	M (2.7)	N (0.345)	L (0.97)	M (3.07)
FATHMM	T (1.69)	D (−3.31)	D (−3.52)	D (−4.02)	D (−2.45)	T (1.3)
PROVEAN	D (−7.84)	D (−4.88)	D (−7.46)	N (−0.32)	N (−0.04)	D (−10.64)
VEST3	0.945	0.402	0.935	0.196	0.264	0.781
MetaSVM	T (−0.278)	D (0.841)	D (1.045)	D (0.181)	T (−0.07)	T (−0.636)
MetaLR	T (0.263)	D (0.862)	D (0.924)	D (0.748)	D (0.561)	T (0.227)
M-CAP	D (0.593)	D (0.905)	D (0.513)	D (0.231)	D (0.098)	D (0.042)
CADD	33	33	33	12.32	23.7	24.8
DANN	0.999	0.999	0.999	0.997	0.999	0.994
fathmm-MKL	D (0.924)	D (0.968)	D (0.985)	N (0.059)	D (0.927)	D (0.928)
**CONSERVATION SCORES**						
GERP++	4.84	2.48	5.42	−0.615	5.65	3.2
phyloP20way	1.048	0.9	1.048	−0.298	1.048	0.964
phastCons20way	0.996	1	1	0	0.983	0.926
SiPhy_29way	13.641	10.434	18.15	3.374	20.078	9.937

*SIFT prediction, D(amaging), T(olerated); Polyphen2 prediction, D (probably damaging), P (possibly damaging); LRT prediction, D(eleterious), N(eutral); MutationTaster prediction, D (disease causing), N (polymorphism); MutationAssessor prediction, functional [H(igh), M(edium)], non-functional [L(ow), N(eutral)]; FATHMM prediction, D(amaging), T(olerated); PROVEAN prediction, D(eleterious), N(eutral); MetaSVM prediction, D(eleterious), T(olerated); MetaLR prediction, D(eleterious), T(olerated); M-CAP prediction, D(eleterious); fathmm-MKL prediction, D(eleterious), N(eutral)*.

### X-Linked Developmental Delay With *ZDHHC9* Mutation

**Figure 1:**
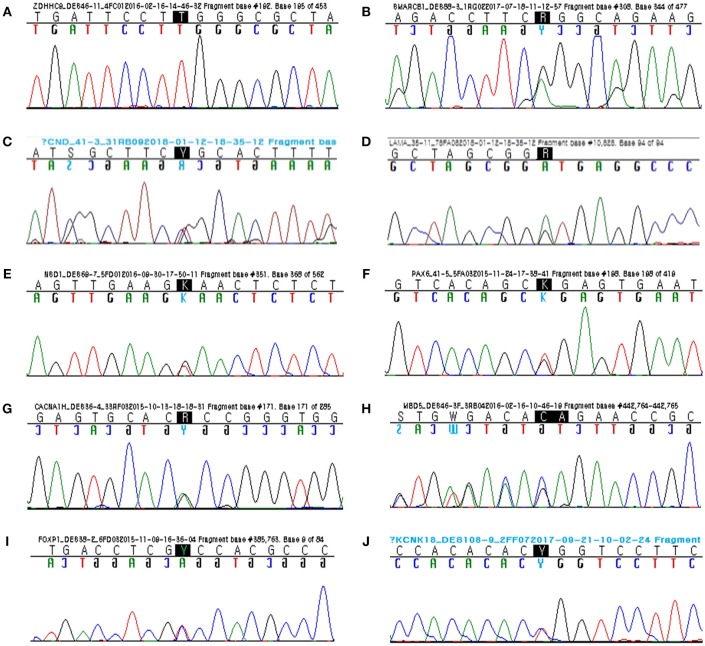
Sanger sequencing confirmed segregation of the rare variants related to the disease phenotypes. **(A)** Hemizygous missense mutation (c.286C>T; p.Arg96Trp) of *ZDHHC9*. **(B)** Heterozygous missense mutation (c.31G>A; p.Gly11Arg) of *SMARCB1*. **(C)** Heterozygous missense mutation (c.4651C>T; p.Arg1551Cys) of *CHD8*. **(D)** Heterozygous donor splice site mutation (c.10828+1G>A) of *LAMA5*. **(E)** Heterozygous nonsense mutation (c.1789G>T; p.Glu866*) of *NSD1*. **(F)** Heterozygous nonsense mutation (c.19G>T; p.Gly7*) of *PAX6*. **(G)** Heterozygous missense mutation (c.5675G>A; p.Arg1892His) of *CACNA1H*. **(H)** Heterozygous frameshift mutation (c.254_255delGA; p.Arg85Asnfs*6) of *MBD5* (reverse). **(I)** Heterozygous missense mutation (c.155C>T; p.Ala52Val) of *FOXP1*. **(J)** Heterozygous missense mutation (c.301T>C; p.Trp101Arg) of *KCNK18*.

A 4-month-old male infant with diagnosis of congenital hypotonia showed weak primitive reflexes such as Moro, grasp, and suckling and laid in a frog-leg position. Obvious DD (cognitive, language, and motor developmental ages of 8–9, 9, and 12 months, respectively) was observed at age of 2 by Bayley-III. His growth parameters at age 2 were 50th percentile for height (50 cm), 75th percentile for head circumference (50.7 cm), and 50th percentile for weight (14.9 kg). Independent walking was achieved at 2.5 years; at age 3, he could say only a few words. By targeted NGS, a hemizygous missense mutation (c.286C>T; p.Arg96Trp) of the *ZDHHC9* gene was identified in the proband. Sanger sequencing confirmed segregation of the *ZDHHC9* mutation with the phenotype and the *de novo* status of the mutation in the proband only, but not in his parents (13) (Figure 1A).

### Coffin-Siris Syndrome With *SMARCB1* Mutation

A 6-month-old female infant showed head lagging and inverted U sign. She did not control her head until 7 months after birth. At 13 months, she was able to sit alone, but did not stand up. She spoke her first words at 16 months. She was admitted to the hospital with frequent infections, including respiratory and urinary tract infections, but her immunological tests were within normal ranges. Her face showed some dysmorphic features: wide nasal bridge, thick eyebrow, long eye lashes, and ptosis. By targeted NGS, a heterozygous missense mutation (c.31G>A; p.Gly11Arg) of the *SMARCB1* gene was identified in the proband. Sanger sequencing confirmed segregation of the *SMARCB1* mutation with the phenotype and the *de novo* status of the mutation in the proband only, but not in her parents (Figure 1B).

### Attention Deficit Hyperactivity Disorder and Intellectual Disability With *CHD8* Mutation

A 13-year-old male with a diagnosis of obesity and ID could say two-word sentences at age 5 years and had behavioral problems. At age 8 years, he underwent neuropsychiatric evaluation and was diagnosed with combined type attention deficit hyperactivity disorder. At age 13 years, he had mild ID (IQ 65) and attended a junior school with additional special education programs. His weight was 94 kg, and body mass index was 35 (both over 97th percentile). By targeted NGS, a heterozygous missense mutation (c.4651C>T; p.Arg1551Cys) of the *CHD8* gene was identified in the proband. Sanger sequencing confirmed segregation of the *CHD8* mutation with the phenotype and the *de novo* status of the mutation in the proband only, but not in his parents (Figure 1C).

### Developmental Delay With *LAMA5* Mutation

A 2-year-old male could not say a single word and was just starting to walk independently. At 2 months, he had no social smile, tracking, or head control. He held his head up at 6 months, rolled over at 9 months, sat independently at 13 months, and stood independently at 23 months. He showed limited interest in objects and other family members. His reactions were slow, and he showed poor eye contact. By targeted NGS, a heterozygous mutation of donor splice site (c.10828+1G>A) of the *LAMA5* gene was identified in the proband (Figure 1D). Sanger sequencing confirmed segregation of the *LAMA5* mutation with the phenotype and the *de novo* status of the mutation in the proband only, but not in his parents. By online bioinformatics tool to predict splicing signals (http://www.umd.be/HSF3/) (14), This splicing mutation was scored as −29.15% by HSF (position weight matrices) and −96.46% by MAxEntScan (maximum entropy) prediction algorithm, respectively (variation threshold: HSF, 10% and MaxEntScan, 30%). Thus, predicted signal was “Broken wildtype donor site” and interpreted as “Alteration of the wildtype donor site, most probably affecting splicing.”

### Sotos Syndrome With *NSD1* Mutation

A 5-year-old male had a triangular face, fronto-temporal sparse hairs, high hairline, prominent forehead, mild micrognathia, high arched palate, large ears, and downward-slanting palpebral fissures. He had owed macro-dolichocephaly, and his growth parameters were 97th percentile for head circumstance (55 cm), 90th percentile for weight (21.4 kg), and > 97th percentile for height (113 cm). He showed behavioral disturbances with deliberate, avoidant, and aggressive behavior. By brain magnetic resonance imaging (MRI), venticulomegaly, modest thinning of the corpus callosum, and prominent extracerebral fluid-filled spaces were found. By audiometry, sensorineural hearing loss (Lt. 6dB, Rt. 40B) was observed. By ophthalmologic examination, amblyopia and hypermetropia were observed. By targeted NGS, a heterozygous nonsense mutation (c.2596G>T; p.Glu866^*^) of the *NSD1* gene was identified (15) in the proband. Sanger sequencing confirmed segregation of the *NSD1* mutation with the phenotype and the *de novo* status of the mutation in the proband and his sibling only, but not in his parents (Figure 1E).

### Aniridia, Autism Spectrum Disorder, and Intellectual Disability With *PAX6* Mutation

A 12-year-old male with a diagnosis of autism-spectrum disorder showed arm flapping, spinning, failure to show interest by pointing, and seizure-like activity. He showed little to no eye contact and did not engage his friends socially. By ophthalmologic examination, a flat-looking iris without structures from the collarette to pupil margin was observed, however his fundi were normal. He underwent brain MRI at age 5, showing normal brain structure and appropriate myelination. By targeted NGS, a heterozygous nonsense mutation (c.19G>T; p.Gly7^*^) of the *PAX6* gene was identified in the proband. Sanger sequencing confirmed segregation of the *PAX6* mutation with the phenotype and the *de novo* status of the mutation in the proband only, but not in his parents (Figure 1F).

### Epilepsy and Intellectual Disability With *CACNA1H* Mutation

A 13-year-old male was referred to our pediatrics for evaluation of ID and epilepsy. He had undergone open heart surgery for a large ventricular septal defect as an infant. He showed complex partial seizure with secondary generalization. Brain MRI showed encephalomalacic change in the right cerebral hemisphere, and we found abnormalities on EEG representing intermittent epileptic discharges in the right temporal areas. His cognitive evaluation revealed a full-scale IQ of 68 (mild ID). By targeted NGS, a heterozygous missense mutation (c.5675G>A; p.R1892H) of the *CACNA1H* gene was identified in the proband. Sanger sequencing confirmed segregation of the *CACNA1H* mutation with the phenotype and the *de novo* status of the mutation in the proband only, but not in his parents (Figure 1G).

### Epilepsy, Autism Spectrum Disorder, and Intellectual Disability With *MBD5* Mutation

A 9-year-female with a diagnosis of severe ID and intractable epilepsy showed a delay in achieving fine/gross motor, language milestones, and social cognition. At 13 months of age, she started to have complex febrile seizures and, at age of 3.5 years, experienced an unprovoked generalized tonic-clonic seizure. Dysmorphic features of face such as broad forehead, hyperterolism, deep nasal bridge, broad nasal root, open mouth, downturned corners of the mouth, and widely spaced teeth were observed. In addition, mild scoliosis, kyphosis, and pes valgus were found on radiological findings. Targeted NGS revealed a heterozygous frameshift mutation (c.254_255delGA; p.Arg85Asnfs^*^6) of the *MBD5* gene in the proband. Sanger sequencing confirmed segregation of the *MBD5* mutation with the phenotype and the paternally inherited status of the mutation in the proband as well as in her father (16) (Figure 1H).

### Autism Spectrum Disorder and Intellectual Disability With *FOXP1* Mutation

A 9-year-old female showed developmental delay, most markedly in the areas of speech and language acquisition. At 20 months, first words appeared, however sentences were not spoken until 4 years. At age of 9, her speech was coherent, but she was not able to hold a conversation. Her speech lacked structure, and she had a very limited vocabulary. In particular, she repeated simple words or phrases and had restricted interests and obsessive behavior traits, based on which she was diagnosed with autism-spectrum disorder. Her cognitive evaluation at 9 years showed a scale of 65. By targeted NGS, a heterozygous missense mutation (c.155C>T; p.Ala52Val) of the *FOXP1* gene was identified in the proband. Sanger sequencing confirmed segregation of the *FOXP1* mutation with the phenotype and the *de novo* status of the mutation in the proband only, but not in her parents (Figure 1I).

### Migraine With Brainstem Aura and Intellectual Disability With *KCNK18* Mutation

A 12-year-old male presented to our emergency department with an acute confused state. He showed agitation, speech impairment, disorientation, and loss of sense of place and time. About 1 h before the mental change, he had experienced severe head pain and dizziness accompanied by vomiting and nausea, aggravated by movement. He had a familial history of migraine. His mother and elder sister had a history of migraine attacks with visual impairment and nausea and mild intellectual disability. At age 10, he was diagnosed with mild intellectual disability (IQ 65), and targeted NGS revealed a heterozygous missense mutation (c.301T>C; p.Trp101Arg) in of *KCNK18* gene in the proband. Sanger sequencing confirmed segregation of the *KCNK18* mutation with the phenotype and the maternally inherited status of the mutation in the proband as well as in his mother (Figure 1J).

## Discussion

This study confirmed the diagnostic value of targeted NGS for investigating the genetic caution of Korean DD/ID with comorbidities. Nine disease-causative variants which contribute to genetic defect of each proband and possible disease-causing variant (c.10828+1G>A of *LAMA5*) were identified in 10 of 35 DD/ID. There are two reports that describe severe neuromuscular transmission failure and/or myopia, facial tics, and failure of neuromuscular transmission in patients with *LAMA5* mutation as homozygous status (17, 18). Further studies are needed to resolve the genotype-phenotype association between DD/ID and *LAMA5*. In other 25 patients, no genetic caution could not be determined because no compelling may be *de novo* variants were identified. We achieved a minimal diagnostic yield of 26% (9/35), which can be increased to 29% (10/35) when and possible disease-causing variant in novel candidate genes are included even though not using a specialized DD/ID-associated gene panel. Similar studies using targeted NGS in patients with ID led to a conclusive diagnostic yield of 17–39% when performed by targeted NGS with ID-associated gene panel only (19–21). Other studies based on whole exome sequencing (WES) (3) or whole genome sequencing (WGS) (22) yielded results not much different from ours.

Targeted NGS can help us to understand the genetic mechanisms of the disease, including the opportunity for targeted interventions, and give us information for future reproductive decisions and genetic counseling. Specifically, Sotos syndrome requires adequate clinical management of the patient, as the clinical outcome and disease course for surveillance are inherently various for this disorder. Similarly, disease-causative variants in genes such as *SMARCB1, PAX6*, and *ZDHCC9* provide information for monitoring of disease-specific secondary complications and thus improving the patient's quality of life. In genes such as *CACNA1H* and *KCNK18*, optional treatment includes channel blockers. Pathological mutations were found in nine of 35 patients, and a likely possible disease-causing variant was found in one patient. Severe DD/ID is mostly of *de novo* origin, and point mutations may be the etiology of most severe DD/ID. By targeted NGS, early diagnosis improves neurological outcomes and can provide psychological relief to families. We suggest that targeted NGS can suggest a genetic diagnosis whose phenotypes include unexplained DD, ID, ADHD, ASD, and epilepsy. In addition, targeted NGS gives more information to diagnose differentially patients with atypical clinical presentations of known genetic diseases. The extensive phenotypic and genetic heterogeneity of DD/ID is the major hindrance for investigating precise molecular diagnosis. Medical and genetic evaluations are intended to identify a particular disorder in order to allow the clinician to provide more information to the parent about the neurological outcomes, comorbidities, and expectation of future requirements. The majority of DD/ID patients remain undiagnosed, and that can have considerable adverse effects for the proband and family members, such as failure to identify proper management, failure to serve anticipatory support and neurological prognosis, failure to recognize the possibility, and the risk of recurrence in future pregnancies (11).

Identification of recurrently possible disease-causing variant along with exact clinical manifestation may help to discover novel DD and/or ID genes and classification of clinical subtypes of DD and/or ID may require specific approaches to medical management. Almost all such mutations show very low prevalence, and their clinical manifestations are often overlooked (3, 23, 24). We carried out family-based targeted NGS using TruSight One Sequencing Panel for patients with DD and/or ID. Candidate variants with potential clinical outcome were also validated using Sanger sequencing as a reference method. Currently, variants are selected for present studies based on report of the affected genes from previous studies and predictive estimations of deleterious effect. A main contribution in exact genetic diagnoses is the detection of patients with different mutations in the same genes, sharing various clinical phenotypes. When screening variants in patients with detailed phenotypic manifestation, few out of more than 5000 diseases in the OMIM database would contribute to clarify the limited category of candidate genes for a certain disease. Moreover, previously established sequence databases are helpful to select candidates of causative variations for a certain disease and to detect the functional significance of discovered variants (25).

Although persuasive for gene discovery, first approaches based on disease phenotype are limited by the differential diagnosis of patients with similar clinical manifestations, which can be difficult for orphan diseases. Differential diagnosis of similarly affected individuals within a family is simple, but familial cases are also uncommon, particularly for autosomal dominant inheritance. In addition, DD/ID more often occurs in the absence of unique phenotypes that can be distinguished prospectively as part of a well-established syndrome, which require an unbiased, genome-wide study to gene discovery. The simultaneous sequencing of trio-WES approach involving both the proband and parents can discover *de novo* mutations located on coding regions in an individual and has promoted rapidly gene discovery. This trio approach to detect *de novo* disease-causing variant is very efficient, because, each individual have one or two *de novo* sequence variants per exome on average. An interesting genetic exploration come out with finding out which *de novo* disease-causing variant is pathogenic, because the majority of *de novo* disease-causing variant of genes detected in cohort-based WES studies are possible candidates for genetic disorder according to evolutionary conservation and/or functional effect. A effective approach to overcome this challenge is to detect genes which are mutated recurrently in the probands with identical or similar phenotype (26). Regardless, singleton study allows identification of novel genes by comparison of sufficient cases in a replication cohort, even though the interpretation of rare non-synonymous variants is unclear and challenging (20). Additionally, it has been reported that genes affected in DD/ID have low intolerance scores; therefore, deleterious variants in these genes tend to occur frequently in the healthy population (27). These genes could be validated by sequencing as part of a diagnostic panel when parents could not be participated for comprehensive analysis of novel causative genes.

On the other hand, more than half of missense variants contributing to disease phenotype were identified (6/10) in this study. With expanding applications of NGS in medical laboratories, increasing rare missense variants are not easily classified as pathogenic or benign. In this situation, the pathogenicity of missense variants of uncertain significance can be estimated by various computational methods based on different predictive algorithms which mainly inferences from evolutionary conservation using protein multiple sequence alignments of the gene of interest and structural features of wildtype and variant proteins. The combinations of different *in-silico* analysis under various conditions, providing tentative guidance for optimal tool selection can be helpful for the identification of candidate genes (28). When prioritizing candidate variants and genes of human diseases, missense variants predicted to be deleterious should be carefully considered. Some missense variants to be pathogenic were actually benign variants, because most computational methods exhibited higher sensitivities than specificities (29). Thus, systematic evaluations regarding its pathogenicity are required to interpret whether the missense variant is involved in human disease. Moreover, allele frequency of the missense variant and mutation rate of a certain gene in the general population should be investigated.

The substantial value of NGS with gene panel for DD and/or ID is that it should enable the application of such molecular testing to larger proportion of patients waiting for genetic diagnosis given the substantially lower cost of NGS, particularly data analysis, interpretation, and storage (30). Gene panel analysis by independent experts determined the medical relevance of such incidental genetic findings, which should generate abundant sequencing data regarding the distribution of mutations and phenotypes related to various genes explained in DD/ID and significantly expand our knowledge of specific conditions related with each gene (19). With targeted gene panel study by NGS, a higher depth of coverage could accomplish this at a lower cost. Increased read depth can promote the detection of small indels that might be passed over by WES. Medical interpretation of novel variants continues challenging, however should become gradually easier with continuous improvement of variant databases of healthy populations along with locus-specific disease databases (31).

However, a number of technical improvements are required to deal with the unequal depth of coverage across the exome, disregarding gaps in coverage, and mapping quality (32). An analysis of referrals for DD and/or ID to our tertiary hospital showed that 71% (5/7) of patients have sporadic DD and/or ID and non-consanguineous parents. One X-linked ID was diagnosed, but the mutation occurred may be *de novo*. This predominance incidence of autosomal dominant inheritance in our study would be even higher if the possible disease-causing variant in candidate genes were included. A total of these mutations arose as eight may be *de novo*, two inheritance, and there was no other possibly disease-causing variant in each proband. Unfortunately, 71% (25/35) of the enrolled patients could not be shown to have a genetic cause by targeted NGS. Broader genomic approaches such as WES or WGS may help to discover clinically relevant disease-causing variant that are not associated to the disease under investigation (33). Expanding to the whole coding region of exons (exome) from a few interesting genes, with the ability to excavate all genes and increasing clinical knowledge related to novel genes with disease pathogenesis, WES or WGS can permit to discover genetic variants that contribute to their hereditary disease, allowing resolution of the diagnostic odyssey and leading potentially to properly medical management through disease-specific therapy (34, 35). However, the actual coverage generated with exome or full-genome strategies is usually insufficient and may result in missed disease-causing variants (22, 36). Moreover, interpretation of variants identified by WES and WGS can be challenging in a clinical setting, because the turnaround time required for validation and characterization of a novel candidate gene is usually too long to be of practical assistance for diagnosing unexplained DD/ID (37). Furthermore, disease-causing variants outside the coding regions, as well as digenic, mosaic, or oligogenic factors leading to DD/ID still remain to be established. These variants may be involved in modifying epigenetic mechanisms such as nucleosomal histone modifications, DNA methylation, and non-coding RNAs expression through regulation of chromatin structure, and post-transcriptional mechanisms without alteration of DNA sequence (38).

In conclusion, targeted NGS through singleton analysis with phenotype-first approaches was able to explain 10 out of 35 DD/ID cases. However, the excavation of plausible genetic causes, may be *de novo*, and X-linked disease-causative variants in DD/ID-associated genes requires further genetic analysis. Prospective study designs for genetic evaluation, referred to as “diagnostic exploration,” should also consider societal medical expenditures, as unsuccessful trials consume limited resources.

## Author Contributions

JH was involved in drafting the manuscript and revising critically for important intellectual content. JJ contributed to the acquisition and interpretation of NGS data. JP made substantial contribution to analysis and interpretation of the data and was involved in drafting manuscript. IL was involved in revising critically for important intellectual content. All the authors read and approved the manuscript for submission.

### Conflict of Interest Statement

The authors declare that the research was conducted in the absence of any commercial or financial relationships that could be construed as a potential conflict of interest. The reviewer IH and handling Editor declared their shared affiliation at the time of review.
